# Complete mitochondrial genome of a leaf-mining beetle, *Podagricomela nigricollis* (Coleoptera: Chrysomelidae)

**DOI:** 10.1080/23802359.2018.1483773

**Published:** 2018-06-26

**Authors:** Xilin Jiang, Qingyun Guo, Jiasheng Xu, Peng Liu, Chengpeng Long, Xiaohua Dai

**Affiliations:** a Leafminer Group, School of Life and Environmental Sciences, Gannan Normal University, Ganzhou, Jiangxi Province, China;; b National Navel-Orange Engineering Research Center, Ganzhou, Jiangxi Province, China

**Keywords:** Podagricomela nigricollis, leaf-mining beetle, mitochondrial genome, phylogenetic analysis

## Abstract

*Podagricomela nigricollis* is a citrus pest that distributes in South China. Currently, there was no complete mitochondrial genome of *Podagricomela* species available in GenBank. Here, we reported the complete circular mitogenome of *P. nigricollis.* It had a total length of 16,756 bp, including 13 protein-coding genes (PCGs), 22 tRNA genes, two rRNA genes, and one A + T-rich region. Among the 13 PCGs, only four (NAD5, NAD4, NAD4l, NAD1) located on the L-strand, whereas the other nine (NAD2, COX1, COX2, ATP8, ATP6, COX3, NAD3, NAD6, COB) located on the H-strand. Phylogenetic analysis using nucleotide sequences of the 13 PCGs indicated that *P. nigricollis* were clustered with six Galerucinae species, which was consistent with previous morphological classification.

The genus *Podagricomela*, belonging to Alticini (Coleoptera: Chrysomelidae: Galerucinae), has 13 species around the world and 10 in China. They are mainly distributed in Oriental Realm (Zhang and Yang 2004). *Podagricomela nigricollis* is an endemic species in South China, feeding on *Citrus* spp. Adult sample was collected from Gannan Normal University (N 25.795, E 114.886), Jiangxi, China, in May 2017. Adult specimen was deposited in the Leafminer Group, School of life and Environmental Sciences, Gannan Normal University. Adults were stored at –80 °C in 100% ethanol. Total genomic DNA was extracted from adult head tissue of *P. nigricollis* using Sangon animal DNA extract kit (Sangon Inc, Shanghai, China). SPAdesv3.9.0 (Bankevich et al. 2012) and A5-miseq v20150522 (Coil et al. 2015) were adopted to construct contigs and scaffolds. The obtained assemblies were analyzed with MUMmer v3.1 (Kurtz et al. 2004) to identify contig mapping. The alignment file was then corrected using Pilon v1.18 (Walker et al. 2014) to get the final mitogenome sequence.

The complete circular mitogenome of *P. nigricollis* has a length of 16,756 bp (Genbank accession number MH325078). The nucleotide composition of *P. nigricollis* mitogenome was biased towards AT 78%, and the total base composition was 41.2% A, 37.6% T, 8.7% G, 12.6% C. The circular genome contained 13 protein-coding genes (PCGs), 22 tRNA genes, two rRNA genes, and one A+T rich region. The order and orientation of the above mitochondrial genes were identical with those of the ancestral insects (Boore 1999). Gene overlaps existed at eight locations with a total length of 108 bp. The length of 13 PCGs was 11,134 bp, with overall base composition of A = 33.4%, T = 43.8%, G = 11.3%, C = 11.5%. All 13 PCGs started with ATN (ATT for NAD2, COX I, ATP8, NAD3, NAD5, and NAD6; ATA for COX II and NAD1; and ATG for ATP6, COX III, NAD4, NAD4L, COB). Twelve PCGs terminated with TAA or TAG, except that NAD4 terminated with an incomplete stop codon T. The longest PCG was NAD5 gene (1713 bp) and the shortest one was ATP8 gene (156 bp). For the 13 PCGs, only four (NAD5, NAD4, NAD4l, NAD1) locate on the L-strand, whereas the other nine on the H-strand. The *rrnL* was 1286 bp long with an A+T content of 81.9% and the *rrnS* was 750 bp long with an A+T content of 82.6%. All of the 22 tRNAs had a typical cloverleaf secondary structure, except for *trnS1* (AGN) whose dihydrouridine arm formed a simple loop. All tRNAs had normal lengths, which varied from 62 to 71 bp.

We downloaded mitochondrial genome sequences of 12 species of leaf beetles (Chrysomelidae) from GenBank, including six species of Galerucinae, four species of Chrysomelinae, and two species of Cassidinae. The phylogenetic tree (Figure 1) were constructed with MEGA 7.0 (Kumar et al. 2016) using maximum likelihood (Stamatakis 2014). The phylogenetic analysis showed that, *Callispa bowringi* (Liu et al. 2018) and *Agonita chinensis* (Guo et al. 2017) formed together as the out-group; *P. nigricollis* clustered with six Galerucinae species, which was consistent with previous morphological classification.

**Figure 1.**
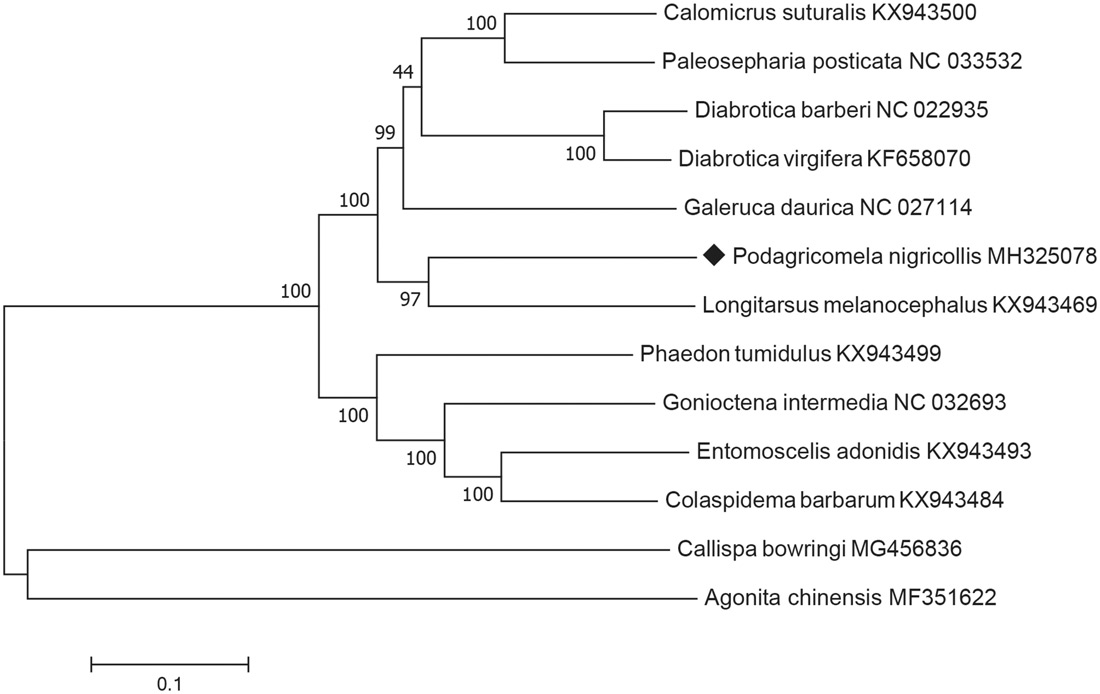
Maximum-likelihood tree based on the concatenated nucleotide sequences of 13 mitochondrial PCGs indicating evolutionary relationships between *Podagricomela nigricollis* and 12 other leaf beetles.
